# Radiation-induced lattice relaxation in $\alpha$-Fe$_2$O$_3$ nanorods

**DOI:** 10.1038/s41598-023-43332-2

**Published:** 2023-09-27

**Authors:** Ahmad M. Khalil, Saad Abdelaal, A. M. Abdelhady, L. I. Abou-Salem, N. M. Shash, Elsayed K. Elmaghraby

**Affiliations:** 1 Physics Department, Faculty of Science, Benha University, Banha, Egypt; 2 Basic Science Department, Faculty of Engineering, Sinai University, Arish, Egypt; 3 Accelerator and Ion Sources Department, Nuclear Research Center, Egyptian Atomic Energy Authority, Cairo, 13759 Egypt; 4 Central Lab for Elemental and Isotopic Analysis, Nuclear Research Center, Egyptian Atomic Energy Authority, Cairo, 13759 Egypt; 5 Experimental Nuclear Physics Department, Nuclear Research Center, Egyptian Atomic Energy Authority, Cairo, 13759 Egypt

**Keywords:** Magnetic properties and materials, Spintronics, Structure of solids and liquids, Nanoparticles, Transmission electron microscopy, Characterization and analytical techniques, Experimental nuclear physics

## Abstract

We report radiation-induced lattice relaxation of the $\alpha$-Fe$_2$O$_3$ and its associated alteration of particle morphology. The $\alpha$-Fe$_2$O$_3$ was grown in solution by microwave hydrothermal synthesis technique in which more than half of the synthesized material was nanorods with axis along the (001) direction. Five sets of the synthesized $\alpha$-Fe$_2$O$_3$ samples were irradiated using gamma-ray from $^{60}$Co cell with doses of 600 kGy, 700 kGy, 800 kGy, 900 kGy, and 1 MGy. The investigation of the pristine and gamma-irradiated samples was carried out using X-ray powder diffraction, transmission electron microscope, and electron paramagnetic resonance methods. Results showed that continuous alternation of radiation-induced lattice compression and expansion causes lattice relaxation. The morphology of the $\alpha$-Fe$_2$O$_3$ nanorods was found to change with absorbed dose into buckyball-shaped particles in response to the alternation of the compression and expansion strain. The EPR results showed a correlation between distortion in the $O_h$–$FeO_6$ octahedron structure and the relaxation of the lattice. The synthesis, growth, and relaxation are discussed in detail.

## Introduction

Iron has many oxidation states from zero-valent metal to Fe(VIII) ion; the configuration of their d-spin-states are determined by the strength of the crystal field. Low-spin states are associated with strong fields while atoms having high-spin states have larger atomic radii. Microwave-assisted hydrothermal synthesis of $\alpha$-Fe$_2$O$_3$ had been reported by many researchers^1–7^. There were many discrepancies among their results considering the morphology and structure of the $\alpha$-Fe$_2$O$_3$ even among those structures synthesized under the same conditions with slight variation a single parameter. Typically, large crystals of $\alpha$-Fe$_2$O$_3$ have a corundum crystal structure with Fe$^{3+}$ ions belonging to d$^5$ configuration and particle buckyball-shaped morphology. The g-value is expected to be near the free-electron value of 2.0023 and is expected to have compressional strain. The $\alpha$-Fe$_2$O$_3$ nanorods, however, are one of the promising morphological structures used in photoelectrochemical processing^8,9^, batteries^10,11^, sensors^12^, shielding^13^ and electrocatalysis^14^. However, the stability of this structure is of utmost concern^15–17^, especially in coordination-assisted dissolution processes^18,19^ and in different environments including the high radiation environment and during electrochemical long-term operation. For example, it is not appreciated to use high-capacity batteries for space application that uses a radiation-sensitive complex. Typically, $\alpha$-Fe$_2$O$_3$ nanorods are not stable as they may coalesce into spherical nanoparticles under the influence of microstrain in the crystal.

Deposition of radiation energy in inorganic oxides, in general, is associated with atom displacement. It is rarely observed that gamma-radiation alters the phase of large oxide crystals of $\alpha$-Fe$_2$O$_3$ due to its large threshold displacement energy^20–22^. However, crystals having the nanometer size may exhibit change in its morphology because the effect of gamma-radiation would depend on the pattern of radiation energy deposition in the substance at the microscopic level of lattice^23^. Most probably, instantaneous stresses could be induced in the lattice due to the formation of a “volume of electrons” having high excitation energy that may or may not be associated with atom displacements^24^.

Crystal strain is usually pronounced in the displacement of some crystallographic planes excluding others. Relaxation of the crystal toward equilibrium strain in all directions is another route for radiation effect in nanometer scale crystals. Hence, post-preparation conditions may cause alteration of the morphology and structure of transition metal complexes and their oxides.

Because the electronic properties of $\alpha$-Fe$_2$O$_3$ depend strongly on the interaction of the Fe$^{3+}$ electronic structure with the surrounding atoms in the crystal field which in turn is dependent on the preparation parameters^25^, the lattice relaxation could be of interact for many electrochemical applications. The investigations were done with the aid of atom coordination techniques including the remnant strain and electron spin resonance.

In the present work, we demonstrate the problem of lattice relaxation or in other words the alteration in the morphology of the $\alpha$-Fe$_2$O$_3$ nanorods, grown along (001) direction, upon exposure to gamma radiation; opening the ground to more scientific research in the field. The radiation-induced lattice relaxation was studied by the X-ray powder diffraction, Transmission Electron Microscope, and Electron Paramagnetic Resonance methods. Because of the nature of ionizing radiation as a consistent effect, the selection of specific radiation doses for the investigation displays only snaps of the particular instants of the whole process. The synthesis, growth, and radiation-induced relaxation are discussed in detail.

## Experimental

### Chemicals and reagents

Chemicals used for preparation of the $\alpha$-Fe$_2$O$_3$ nanorods were: FeCl$_{3}$–6H$_{2}$O from Sigma-Aldrich CAS Number: 10025-77-1 and acid-free, NH$_{4}$OH (CAS number 6699-20-3, and deionized water with the resistivity close to 18 M$\Omega$cm was used. Laboratory-grade ethanol was used as an assisting agent with water for sample washing and dispensing.

### Preparation of hematite

The $\alpha$-Fe$_2$O$_3$ nanorods were prepared by microwave-assisted hydrothermal method, *cf*. Ref^26^. Two separate solutions were prepared prior to mixing, FeCl$_{3}$ solution was prepared by dissolving an amount of 0.6 moles of FeCl$_{3}$ 6H$_{2}$O in 100 ml of deionized water while the ammonium hydroxide solution was prepared from 1.8 moles of NH$_{4}$OH in 50 ml of deionized water. The two solutions were mixed slowly by placing the beaker containing iron chloride solution under stirring conditions and the NH$_{4}$OH solution added in a dropwise manner. The pH of the mixed solution starts to increase to about pH 11 and is transferred to a 120 ml microwave-ready Teflon container having a pressure cap. The microwave reactor was ETHOS, USA, and works at a frequency of 2.45 GHz at the maximum output power of 1200 W. The microwave heating time was 15 min. Directly afterward Teflon container was set to ventilation mode (without delay); the containers were kept for 10 min in ventilation mode and then transferred for gradual cooling at room temperature. The precipitates were filtered using 0.45 $\mu$m Whatman filter paper and then washed with 500 ml of 1:1 mixture of deionized water and ethyl alcohol. A vacuum drying system was used to dry the remaining powder. This process synthesizes the pristine $\alpha$-Fe$_2$O$_3$.

### Gamma irradiation

Five sets of the dry $\alpha$-Fe$_2$O$_3$ nanorods samples were irradiated using gamma-ray from $^{60}$Co cell at an Egyptian Atomic Energy Authority facility with a dose rate of about 1.1 kGy/h. The gamma cell ensures even exposure by rotating the samples during irradiation. Due to the long irradiation time needed to reach doses of 600 kGy, 700 kGy, 800 kGy, 900 kGy, and 1 MGy, the accumulated radiation dose was calculated using^27^:

1 $$\begin{aligned} D=D_o \frac{\big (1-e^{-\lambda \Delta t_i}\big ) e^{-\lambda (t-t_o)}}{\lambda } \end{aligned}$$

where $D_o$ is the dose rate of the gamma cell at the time of calibration ($t_o$), *t* is the time of measurements, and $\Delta t_i$ is the time interval of irradiation. The decay constant of $^{60}$Co is $\lambda$ = 0.1315 y$^{-1}$. Samples were kept in a polyethylene container warped with aluminium foils after irradiation until the characterizations were carried out.

### Characterization

#### X-ray diffraction

The X-ray powder diffraction (XRD) method was used to collect information considering the crystal structure using the SHIMADZU X-ray diffractometer at the 40 keV acceleration and current of 30 mA. The 1.5406 Å Cuk$_\alpha$ radiation was filtered using a 10 $\upmu$m nickel filter. The scanning parameters were adjusted as follows: The slit width was 1$^{\circ }$, the scatter slit width of 1$^{\circ }$, the receiving slit width of width 0.15 mm, the scan range was 4–90$^{\circ }$ with scan speed 8$^{\circ }$ /min, and the sampling pitch of was 0.02$^{\circ }$. Noise due to the small size of the samples and their nanometer morphology were unavoidable and are treated with smoothing of the original data.

#### Electron paramagnetic resonance

The electron paramagnetic resonance spectroscopy (EPR) was done by X-band microwave using Bruker’s EMX spectrometer. To avoid the effect of the low temperature on the $\alpha$-Fe$_2$O$_3$ lattice and its morphology, the measurements were performed at room temperature. Measurements were performed directly after irradiation. A standard Bruker’s rectangular cavity (Model ER-4102 cf. Ref.^28^) was used. The klystron power was kept at 1 mW at frequency $\nu _M$ = 9.717 GHz.

#### Transmission electron microscope

The morphologies of the material were observed using transmission electron microscope (TEM) Model JEM-2100 (JEOL, Tokyo, Japan). Specifically for the TEM imaging needle-tip portion of each sample was dispensed in 1 ml of ethanol under the ultrasonic influence for at least 10 min to force the stuck particle to separate from each other. One drop of the ethanol solution was taken while the ultrasonic power was still operative and placed on the C-coated copper grid of the TEM apparatus. The TEM parameters were adjusted as follows: electron acceleration of 200 kV in bright field mode.

### Uncertainties

Experimental uncertainties were only due to the statistical nature of photon detection in XRD. The uncertainties propagate through consecutive steps of calculations and were determined using the Provisional Rule^29^, taking into account the independencies among these sources of deviation and adding in quadratic error propagation.

## Results and discussion

### Identification of the oxide phase

**Figure 1:**
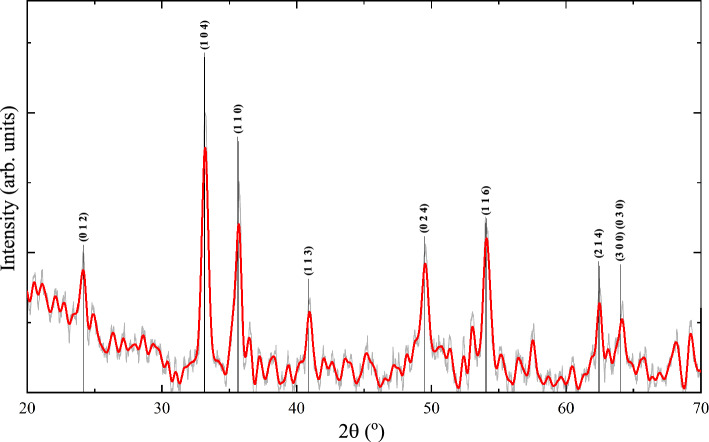
The XRD diffractogram of the as-prepared sample. The most obvious crystallographic plans (012), (104), (110), (113), (024), (116), (214), (300), and (030) identified reflection corresponds to rhombohedral $\alpha$-Fe$_2$O$_3$ crystal system from ICDD JCPDS card No. #72-0469. The light grey line shows the data before smoothing. Natural noise is due to the small size of the samples. Noise due to the small size of the sample and its nanometer morphology were unavoidable and are treated with smoothing of the original data.

**Figure 2:**
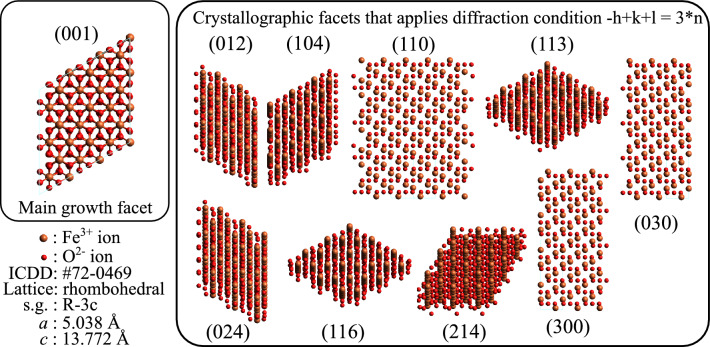
The discussed crystal facet of $\alpha$-Fe$_2$O$_3$ , plotted using Avogadro, an open-source molecular builder and visualization tool^30^ using ICDD JCPDS card No. #72-0469 structural data.

The XRD diffractogram of the as-prepared (pristine) sample showed the formation of $\alpha$-Fe$_2$O$_3$ phase of the iron oxide, see Fig. 1. The identified reflection corresponds to rhombohedral $\alpha$-Fe$_2$O$_3$ crystal having space group R-3c (No. 167) with cell parameters: *a* = 5.038 Å, *c* = 13.772 Å. The cell volume was reported from ICDD JCPDS card No. #72-0469 to be 302.72 Å$^3$. The crystallographic plans (012), (104), (110), (113), (024), (116), (214), (300), and (030) fulfill the condition $-h$+*k*+*l* = 3 *integer* of the diffraction. These planes together with (001) plane are illustrated in Fig. 2. A list of $d_{hkl}$ spacing is given in Table 1. The (001) plane does not fulfill the diffraction condition of the rhombohedral lattice.

**Table 1:** The interplanes spacing ($d_{hkl}$) in pm for the observed diffraction pattern in Fig. 1.

Plane	$d_{hkl}$	$\theta _{hklh^{*}k^{*}l^{*}}$ in deg.							
(*h* *k* *l*)	(pm)	(0 1 2)	(1 0 4)	(1 1 3)	(0 2 4)	(1 1 6)	(2 1 4)	(1 1 0)	(3 0 0)
(0 0 1)		57.6	38.3	61.2	57.6	42.3	64.4	90	90
(0 1 2)	368.8		47	26	0	27.3	36.2	43	65
(1 0 4)	269.8			32	29.9	19.7	29.9	57.5	51.7
(1 1 3)	220.4				26	18.9	10.2	28.7	40.6
(0 2 4)	169.5					27.3	36.2	43	65
(1 1 6)	148.6						23.7	47.7	54.3
(2 1 4)	145.1							27.7	31.5
(1 1 0)	251.6								30
(3 0 0)	145.1								

The angle between crystallographic directions ($\phi _{hklh^*k^*l^*}$) is expressed in degree.

### The $\alpha$-Fe$_2$O$_3$ morphology

**Figure 3:**
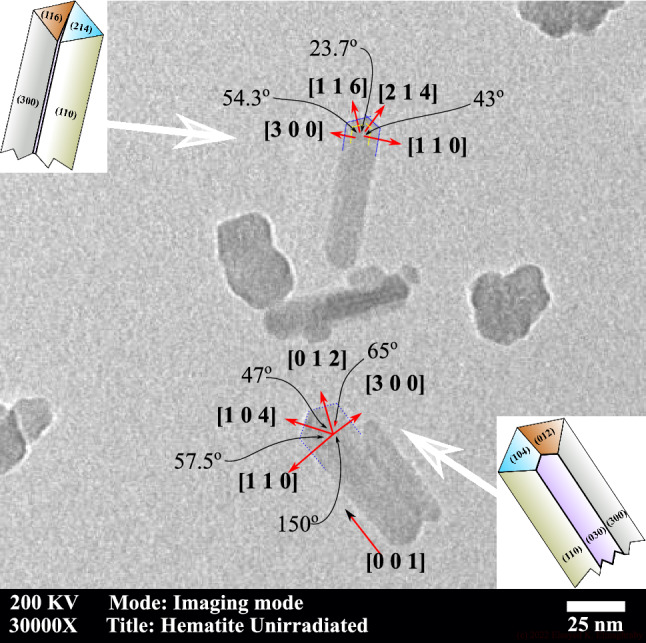
Bright-field TEM image of $\alpha$-Fe$_2$O$_3$ nanorods. Image is the X30000 magnification from the corresponding image in Fig. 10. Bold lines (red on-line) represent the crystallographic directions. The dotted line shows the outlines of the $\alpha$-Fe$_2$O$_3$ nanorods (blue on-line). The two inserts show a schematic diagram of the facets of the $\alpha$-Fe$_2$O$_3$ nanorods analyzed in this TEM image. The angles between crystallographic directions are expressed in degrees.

Previous investigations on the XRD and TEM had suggested the growth of $\alpha$-Fe$_2$O$_3$ nanorods^31^ or nanotubes^18,19^ along the [001] direction. In the present work, the bright field image was used to inspect the shape and morphology of the as-prepared $\alpha$-Fe$_2$O$_3$. Figure 3 shows the morphology of the pristine sample as identified using TEM with X30000 magnification. The morphology showed a set of nanorods bearing a polygonal outline of smooth facets. The dimensions of the rods were between 15 nm and 22 nm in diameter and 60 nm and 75 nm in length. Typically, there were buckyball-shaped particles with a diameter of about 25 nm. The two shapes coexisted in all TEM images of the pristine sample but with different proportions.

There are polyhedral shapes at the top and side of the nanorods which suggests its facets can be identified by correlation with inclusion angles. The angles between outlines of the polygonal shape at the top of two nanorods were determined using ImageJ free software^32^. The angle between crystallographic direction was determined for the approved $\alpha$-Fe$_2$O$_3$ rhombohedral structure using the formula

2 $$\begin{aligned} \cos (\phi )&= \frac{hh^*+kk^*+\frac{1}{2}(hk^*+kh^*) +\frac{3}{4}\frac{a^2}{c^2}ll^*}{gg^*} \end{aligned}$$

3 $$\begin{aligned} g&=  \sqrt{\left( h^{2}+k^{2}+hk+\frac{3}{4}\frac{a^2}{c^2}l^{2}\right) } \end{aligned}$$

4 $$\begin{aligned} g^*&=  \sqrt{\left( h^{*2}+k^{*2}+h^*k^*+\frac{3}{4}\frac{a^2}{c^2}l^{*2}\right) } \end{aligned}$$

Remembering that the inclusion angles between planes are the complementary angle ($180^\circ -\phi _{hklh^*k^*l^*}$) between their corresponding direction and with the aid of the list of angles in Table 1, we had eliminated the far possibilities in assigning the crystal facets. The assigned plane direction was illustrated by the bold line in Fig. 3 and its insert. The dashed line in Fig. 3 represents the expected center line of the facets. The results showed that the direction of the growth of the rods shall be the [001] direction in agreement with Refs.^18,33–37^. The top facets of the *tetrahedral shape* (not to be confused with tetrahedral position of iron ion, $O_h-FeO_6$) at the top of the rod shall be the (116), (104), (012), and (214) planes. It is evident that the rhombohedral structure in Fig. 3 support the results of the crystal structure of the $\alpha$-Fe$_2$O$_3$ obtained from XRD in Fig. 1.

### The [001] growth

Using the current preparation parameters, the $\alpha$-Fe$_2$O$_3$ growth proceeds mostly by deposition of Fe$^{3+}$ ions on the (001) planes of the $\alpha$-Fe$_2$O$_3$, see left panel of Fig. 2 and Table 1. In order to comprehend such a growth mechanism, the surface reactivity through Stern layer formation was considered. The (001) facet is terminated with oxygen atoms that are doubly coordinated with subface iron atoms($\equiv$Fe$_2$–O), i.e. the attached –OH group from the solution is located above the mid-distance between two Fe atoms, this makes the surface density of OH groups around 13.7 nm$^{-2}$ as illustrated in Table 2.

**Table 2:** The number density of the OH groups of $\alpha$-Fe$_2$O$_3$ in aqueous solution for various facets of the crystal.

Plane	d (pm)	Number density OH groups (nm$^{-2}$)			
		$\equiv$Fe–OH	$\equiv$Fe$_2$–OH	$\equiv$Fe$_3$–OH	Total
(0 0 1)		0	13.7	0	13.7
(0 1 2)	368.8	7.3	0	7.3	14.6
(1 0 4)	269.8	5.3	5.3	5.3	15.9
(1 1 3)	220.4	4.1	4.1	8.3	16.5
(1 1 0)	251.6	5	5	5	15

The coordination may be singly coordinated ($\equiv$Fe–OH), doubly coordinated ($\equiv$Fe$_2$–OH), or/and triplly coordinated ($\equiv$Fe$_3$–OH). Data values are from Ref.^38^. The densities are given in nm$^{-2}$.

On the other hand, other facets of the $\alpha$-Fe$_2$O$_3$ crystal, i.e. (012), (104), (110), (113), (024), (116), (214), (300) and (030), are singly coordinated (the attached OH group is located above Fe atom), doubly coordinated, or triply coordinated (the attached OH group is located above the center of the triangle connecting three Fe atoms) surfaces^38^, see right panel of Fig. 2 and Table 2. The total densities of the (012), (104), (113), and (110) facets are 14.6 nm$^{-2}$, 15.9, nm$^{-2}$ 16.5 nm$^{-2}$, and 15 nm$^{-2}$, respectively.

Zhong et al.^37^ showed the correlation between the formation of the radicals and the morphology of the synthesized compounds of iron during the microwave-assisted hydrothermal synthesis method. The synthesis route may proceed through the formation of the $\alpha$-FeOOH^39^ to induce the growth of the rhombohedra $\alpha$-Fe$_2$O$_3$ , in particular, along the [001] direction to form nanorods as proposed in Ref.^37^. The basel (001) plane is formed by both Fe and O atoms. The reaction between FeCl$_{3}$ and NH$_{4}$OH is initiated instantaneously forming FeOOH precipitates which rises the solution temperature to about 40 $^\circ$C during the precipitation process according to the reaction in Eq. 5. However, dropwise addition ensures the formation of Fe(OH)$_3$ which slowly transforms from ferrihydrite to phases of FeOOH according to the reaction in Eq. 6^34^.

5 $$\begin{aligned} &  Fe^{3+} + 3 OH^{-} \rightarrow Fe(OH)_3 \end{aligned}$$

6 $$\begin{aligned} &  Fe^{3+} + 3OH^{-} \rightarrow FeOOH + H_2O  \\ &  Fe(OH)_3 \underset{\triangle }{\overset{NH_4OH}{\longleftrightarrow }} FeOOH + H_2O \end{aligned}$$

The FeOOH may be any phase between $\alpha$ and $\gamma$ phases of FeOOH. But mostly $\alpha$-FeOOH. According to the basics of the microwave-assisted hydrothermal synthesis method, heat is generated in the reaction pile with the aid of the interaction of the 2.45 GHz radiation with water molecules by dipole polarization or ion conduction of water molecules. The basic aspect of the ability of microwave-assisted hydrothermal synthesis is the radiation penetration depth which covers the entire pile of the chemical reactor simultaneously and in turn, avoids the temperature gradient that could affect the preparation reaction size and morphology.

In the microwave-assisted hydrothermal synthesis technique, $\alpha$-FeOOH would first be synthesized and then transformed into $\alpha$-Fe$_2$O$_3$ by dehydration in the microwave-assisted hydrothermal synthesis treatment. The $\alpha$ and $\gamma$ phases of FeOOH are those seeds for the formation of $\alpha$-Fe$_2$O$_3$^40^, see Eq. 7

7 $$\begin{aligned} 2 \alpha -FeOOH&\underset{\triangle }{\overset{}{\longleftrightarrow }}&\alpha -Fe_2O_3 + H_2O  \\ 2 \gamma -FeOOH&\underset{\triangle }{\overset{}{\longleftrightarrow }}&\gamma -Fe_2O_3 + H_2O  \\ \gamma -Fe_2O_3&\underset{\triangle }{\overset{}{\longleftrightarrow }}&\alpha -Fe_2O_3  \\ 2 \gamma -FeOOH&\underset{\triangle }{\overset{}{\longleftrightarrow }}&\alpha -Fe_2O_3 + H_2O \end{aligned}$$

The OH groups are proton-reactive species, the increase in their surface density indicates more reactivity of the surface. In solution, ions with the sign of charge opposite to that of surface, usually dissociated hydrogen, are accumulated at the surface, i.e. the facet is suppressed by the positive charge, in what is known as “Stern layer”^41,42^, the electrical double layer made of positive ions on the surface and the counter-ions attracted to the surface charge, electrically screening the first layer. The Stern layer prevents Fe$^{3+}$ ions from reaching the facets in a manner exponentially proportional to the surface concentration of the ion pairs^38,43^. That makes the growth along the [001] direction more favorable for the growth than other crystallographic directions because of its lower number density of OH group, see Table 2.

It is worth mentioning that the Ostwald Ripening Mechanism (ORM) (cf. Ref.^35^) does not explain the growth of hematite into nanorods nor the effect of radiation. For the moment, the mechanism of transformation from FeOOH to $\alpha$-Fe$_2$O$_3$ through the ORM involves atoms relocation, i.e. the dissolution of small crystals or sol particles and the re-deposition of the dissolved species on the surfaces of larger crystals or sol particles according to IUPAC^44^. The ORM occurs because larger particles are more energetically favored than smaller particles due to the diffusion of dispersed phase atoms through the continuous phase. This claim was based on the crystal structural analogy between $\alpha$-FeOOH and $\alpha$-Fe$_2$O$_3$ which was claimed to make the phase transformation energetically possible according to Cudennec and Lecerf^40^. However, in the growth into rods and wires, ORM does not seem the appreciated mechanism because the growth always proceeds along the direction of the smaller (001) facet.

### The effect of ionizing radiation

#### Electron paramagnetic resonance study

**Figure 4:**
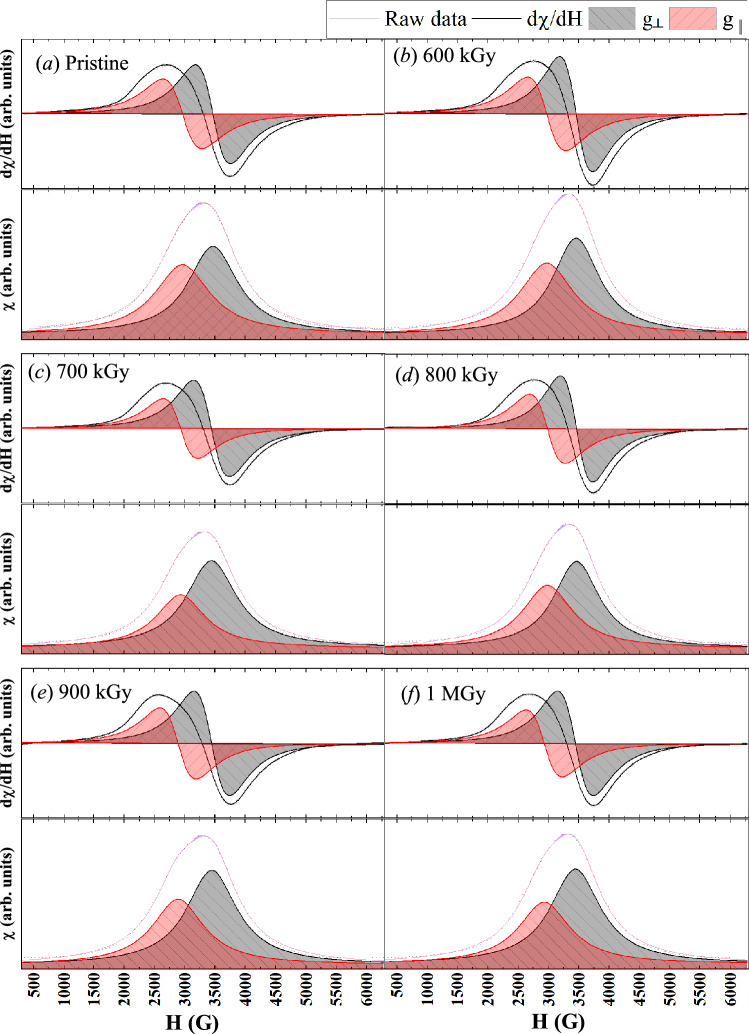
The EPR spectra recorded at room temperature for the unirradiated pristine $\alpha$-Fe$_2$O$_3$ nanorods (sub-figure **a**) together with the $\gamma$-irradiated samples (sub-figures **b** for 600 kGy, **c** for 700 kGy, **d** for 800 kGy, **e** for 900 kGy, and **f** for 1 MGy). The absorption spectra (indicated by susceptibility $\chi$) are illustrated at the bottom of each sub-figure while its differential ($d\chi /dH$) is plotted above it, both are in arbitrary units and against the magnetic field strength (H) in the unit of Gauss (1 mT = 10 G). Raw data are illustrated by thin gray lines while its smoothed line is used for analysis. The two shaded areas in each figure represent the Lorentzian peak fitting to separate the parallel ($g_\parallel$) and perpendicular ($g_\perp$) components of the microwave absorptions.

In EPR spectroscopy, the microwave radiation frequency is held constant at 9.717 GHz while the magnetic field is varied from the lowest possible value to about 10000 G. The magnetic field causes Zeeman splitting of the possible energy levels so that the absorption of microwave occurs when its energy equals the energy difference induced by the magnetic field. The absorption spectra and their differential are shown in Fig. 4, both are against the magnetic field strength (H) in Gauss. At the condition of resonance, Landé g-value is related to magnetic field strength through the relation^26,27^;

8 $$\begin{aligned} g\text {-value} =\frac{h{{\nu }_{M}}}{{{\mu }_{B}}{{H}_o}}, \end{aligned}$$

where $H_o$ is the magnetic field strength at the resonance in the units of Tesla (1 T = 10000 G) and shall be replaced by the $H_{o1}$ and $H_{o2}$ to obtain $g_\parallel$ and $g_\perp$, respectively. Here, the microwave frequency is $\nu _M=9.717\times 10^9$ Hz, the Bohr magnetron is ${\mu }_{B}=9.27402\times {10}^{-24}$ J/T, and the Planck’s constant $h=6.62607\times 10^{-34}$ J s.

**Figure 5:**
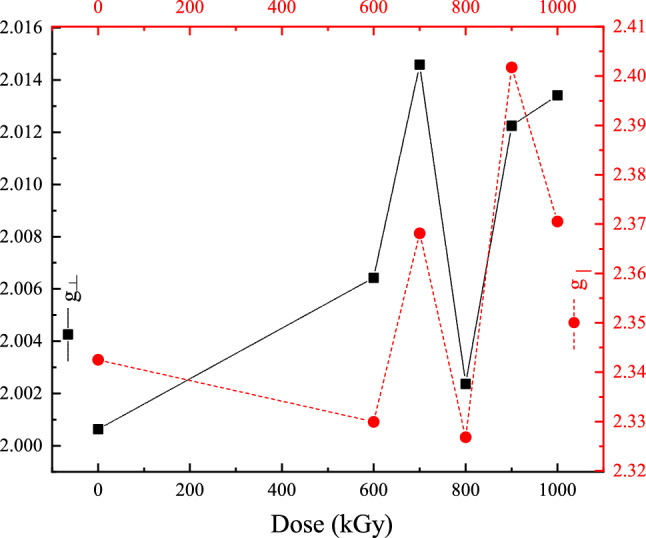
The variation of contributions of the parallel ($g_\parallel$ on the right axis labels) and perpendicular ($g_\perp$ on the left axis labels) with radiation dose.

It is known that the EPR spectra at elevated concentrations of the d-electrons are often characterized by broadened absorption lines^45^ which makes it difficult to determine the resonance frequencies. In the present work, and to obtain the exact values of the resonance frequencies (denoted $H_{o1}$ and $H_{o2}$), each absorption spectra was fitted using a double Lorentzian peak profile to separate the contributions of the parallel ($g_\parallel$) and perpendicular ($g_\perp$) absorptions, then the first derivative of each component was used to obtain this corresponding Lande g-value. The corresponding resonance parameters are given in Table 3 and Fig. 5.

**Table 3:** Fitting parameters for EPR spectra in Fig. 4 and their corresponding g-values.

Dose	$H_{o1}$	$H_{o2}$	$g_\perp$	$g_\parallel$
(kGy)	(G)	(G)		
0	3467	2961	2.0006	2.3425
600	3457	2977	2.0064	2.3299
700	3443	2929	2.0146	2.3681
800	3464	2981	2.0024	2.3268
900	3447	2888	2.0123	2.4017
1000	3445	2926	2.0134	2.3705

The EPR spectrum of the pristine and irradiated samples exhibited an intense resonance signal at the range 3443 G to 3467 G and weaker resonance at the range 2826 G to 2988 G. The corresponding g-values are from 2.0006 to 2.0146 and from 2.3268 to 2.4017, respectively. There were fluctuations in the value of $g_\parallel$ or $g_\perp$ if seen individually as the dose increase as illustrated in Fig. 5; but the values of $g_\parallel$ and $g_\perp$ vary with each other consistently, i.e. increase or decrease with each other.

**Figure 6:**
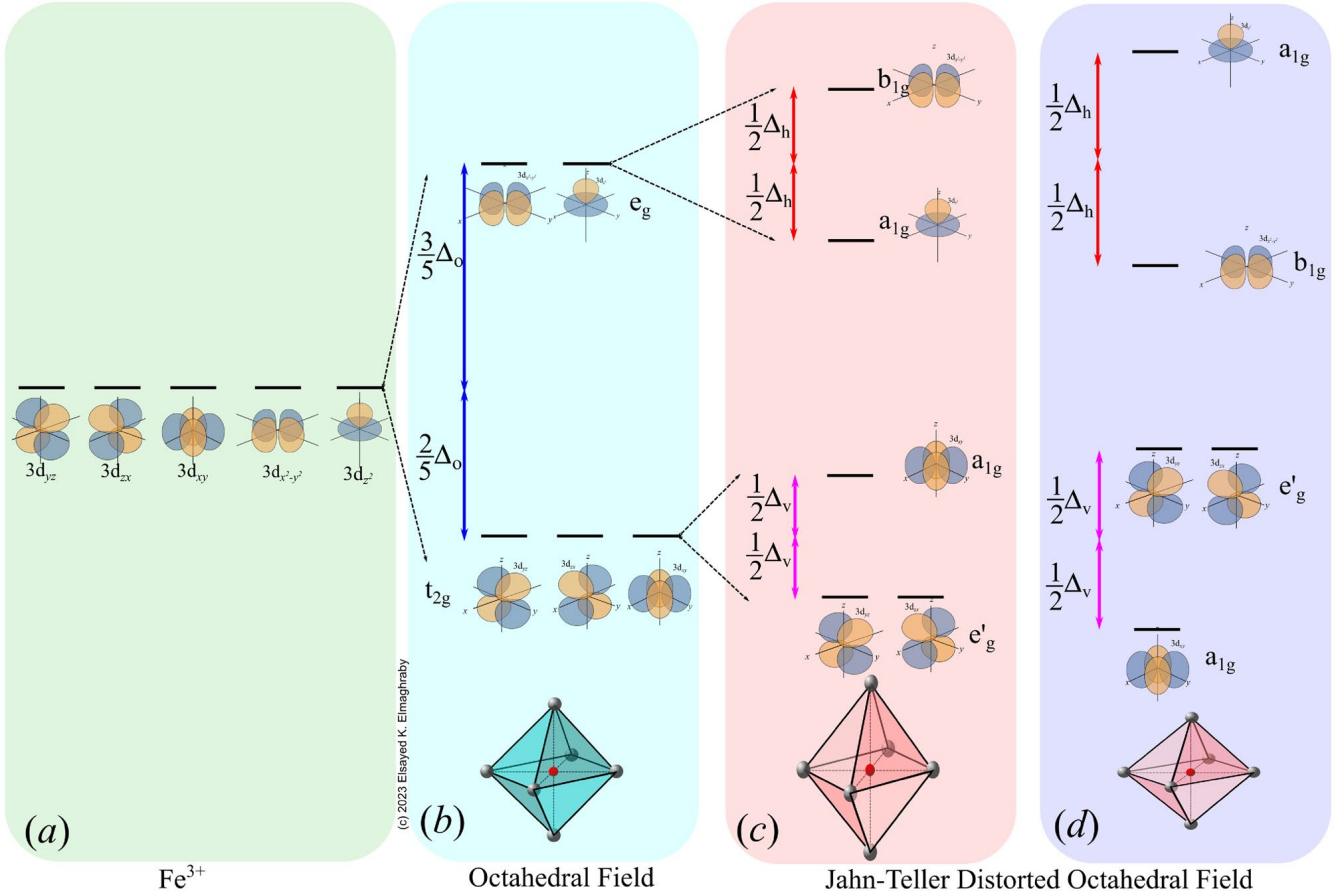
The energy level configuration of the d$^5$ under different coordinations. (**a**) spherical charge symmetry, (**b**) perfect octahedron symmetry, (**c**) z-axis expansion distorted octahedron, and (**d**) z-axis compressed octahedron.

In order to interpret the reason for the two resonances and their variations it must be known that isolated transition metal ion, or alternatively ion having a symmetrical field in all directions, the energies of all five *d* orbitals would degenerate^46^. These levels are equienergetic, see Fig. 6a. In $$\alpha $$-Fe$$_2$$O$$_3$$ crystal, the Fe$$^{3+}$$ and six of the O atoms are repeated in a unit of the form octahedron $$O_h$$-$$FeO_6$$ unit. This simple octahedral crystal field, if stable, exhibits level splitting, Fig. 6b. The $$d_{x^2-y^2}$$ and $$d_{z^2}$$ experience larger potential energy and, consequently, increase in their energy by value $$\frac{3}{5}\Delta _o$$. Inverse causes reduce the energy of the $$d_{xy}$$, $$d_{yz}$$, and $$d_{zx}$$, which reduced their energy by value $$\frac{2}{5}\Delta _o$$. Here, $$\Delta _o$$ is the splitting energy. Hence, the coordinated oxygen group produces $$e_g$$ doublet and triply-degenerate $$t_{2g}$$ symmetric orbitals with corresponding antibonding states of $$e_g^*$$ and $$t_{2g}^*$$^47^. The electron configurations of the ion comprises $$(t_{2g})^{5-n}(e_g)^n$$, where $$1< n < 4$$^48^. The lowest spin configurations are S=1/2 (i.e. $$(d_{xz}d_{yz})^4(d_{xy})^1$$ or $$(d_{xy})^2(d_{xz}d_{yz})^3$$ )^49^.

Jahn and Teller^50^ showed that any nonlinear molecule can not be stable in the d degenerate electronic state and the orientation shall be distorted to lower the degree of symmetry in order to break such degeneracy. In the $O_h$-$FeO_6$ structure with five *d* electrons ($d^5$), the Jahn–Teller effect is more pronounced. For $\alpha$-Fe$_2$O$_3$, distortion of the oxygen octahedron gives rise to distortion in the crystal field. The special cases of the distortion are the “symmetrical” distortions of elongation (Fig. 6c) and contraction (Fig. 6d) along the z-axis. The octahedron distortion causes further splitting of $e_g$ and $t_{2g}$ energy states depending on the type of distortion. As a result, the $e_g$ doublet splits into $a_{1g}$ singlet and $b_{1g}$ singlet while and the triply-degenerate $t_{2g}$ orbital splits into $e_{g}^\prime$ doublet and $a_{1g}$ singlet^49,51–54^. Technically, it is difficult to determine *a priori* which of these split orbitals has the lowest energy^55^; strain analysis with XRD shows only the existence of strain relative to equilibration octahedron.

Based on these facts and due to the consistency between the variation of $g_\parallel$ and $g_\perp$ with radiation dose, there is an indication of the existence of a relaxation phenomenon triggered by the radiation energy deposition in the synthesized nanorods.

#### X-ray study

**Figure 7:**
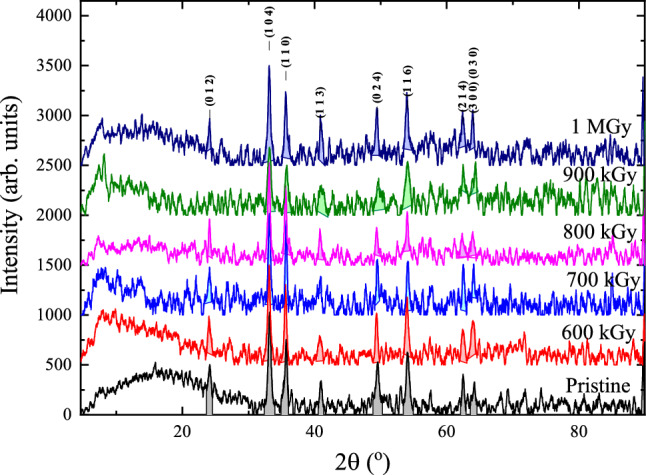
The XRD diffractograms of the Pristine and gamma-irradiated $\alpha$-Fe$_2$O$_3$ . The crystallographic plans (012), (104), (110), (113), (024), (116), (214), (300), and (030) identified reflection corresponds to rhombohedral $\alpha$-Fe$_2$O$_3$ crystal system from ICDD JCPDS card No. #72-0469. The baselines are shifted by 500 cps in every subsequent diffractogram for better illustration. Noise due to the small size of the samples and their nanometer morphology were unavoidable. Shaded areas represent the fitting to the Lorentzian line-shape profile.

The XRD diffractograms of the gamma-irradiated $\alpha$-Fe$_2$O$_3$ are illustrated in Fig. 7 in comparison with the XRD diffractograms pristine $\alpha$-Fe$_2$O$_3$ sample. The alpha phase of the $\alpha$-Fe$_2$O$_3$ persists for different radiation doses but the intensity and the broadening of the diffraction peaks differ.

**Figure 8:**
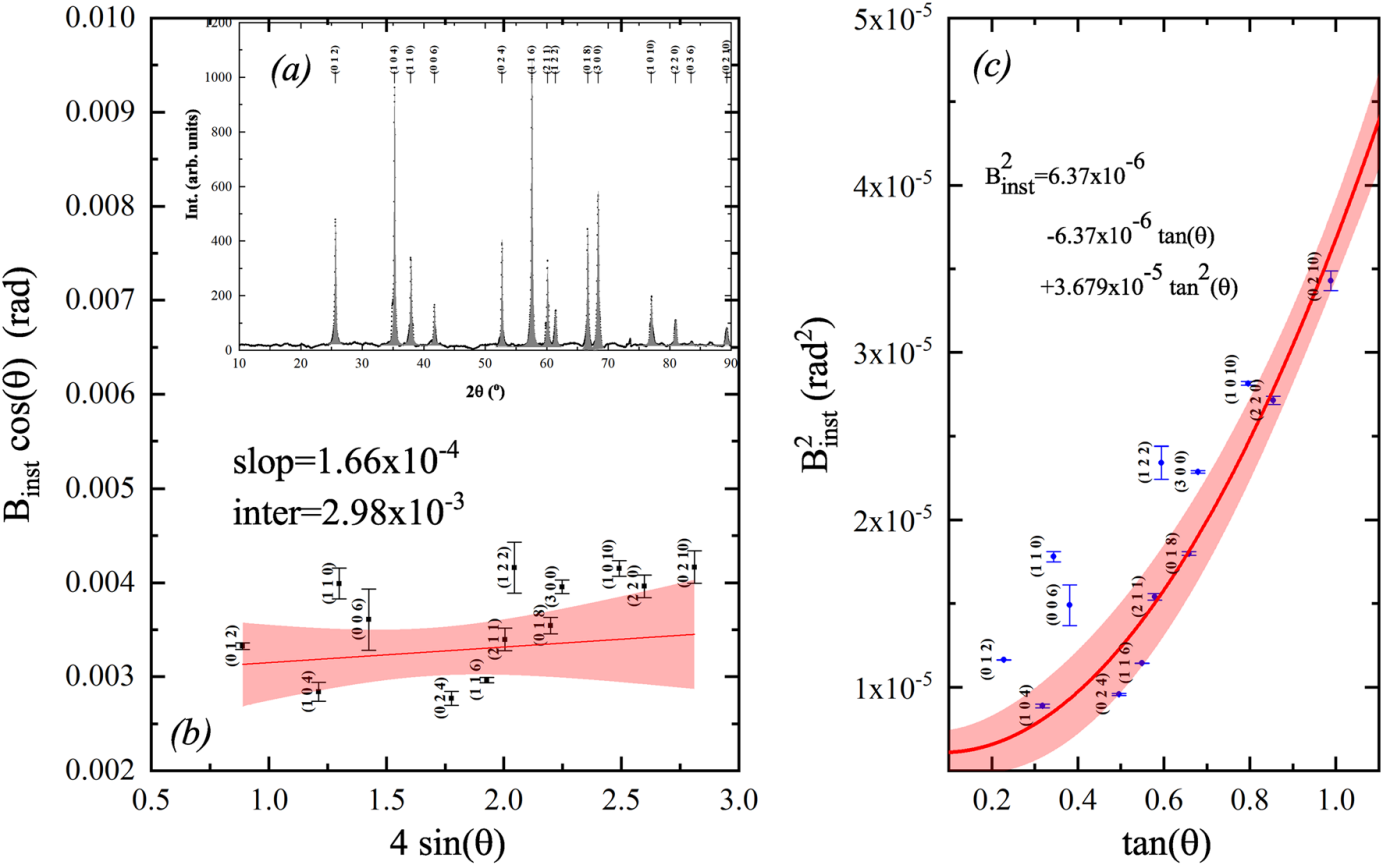
(**a**) XRD diffractogram of reference 50 $\mu$m $\alpha$-Al$_2$O$_3$ particles. (**b**) Williamson–Hall plot for the X-ray diffraction of $\alpha$-Al$_2$O$_3$ reference 50 $\upmu$m powder. (**c**) Semi-empirical fitting using the Cagliotti equation.

In order to explain the variation with absorbed dose, the broadening of the diffraction peaks was calculated after fitting to the Lorentzian line-shape function of the form:

9 $$\begin{aligned} I_{hkl}=I_{0,hkl} + \frac{2A_{hkl}}{\pi }\frac{W_L}{4(2\theta -2\theta _{hkl})^2+W_L^2} \end{aligned}$$

where $A_{hkl}$ is the peak area, $I_{0,hkl}$ is the baseline intensity, $2\theta$ is the angle, $2\theta _{hkl}$ is the diffraction peak position, and $W_L$ is the Lorentzian peak width. The values of peak broadening, of the specimen (${{B}_{\text {specimen}}}$) was obtained by subtracting the instrumental peak broadening (${{B}_{\text {inst.}}}$) from the peak broadening of the sample (${{W}_{L}}$). The values of the instrumental broadening are obtained by measuring the X-ray diffraction broadening of a 50 $\mu$m Corundum ($\alpha$-Al$_2$O$_3$) particles. Corundum was used because of is similarity with $\alpha$-Fe$_2$O$_3$ in the rhombohedral structure, see Fig 8a. The peaks broadening was also determined using the Lorentzian line shape in Eq. 9. Because of the large dimension of the $\alpha$-Al$_2$O$_3$ particles, only the instrumental broadening appears in the diffraction pattern with a small contribution of the strain broadening. The condition for successful results is the $\alpha$-Al$_2$O$_3$ reference powder should be free from strain as possible. For confirmation, the Williamson–Hall plot for the x-ray diffraction of $\alpha$-Al$_2$O$_3$ reference 50 $\mu$m powder was obtained as in Fig. 8b. The Williamson-Hall plot fulfills the relation

10 $$\begin{aligned} B_{hkl}\cos \theta = \frac{0.95 \lambda }{D_{hkl}}+ 4 \varepsilon _{hkl} \sin \theta _{hkl} \end{aligned}$$

where $B_{hkl}$ is the broadening whether instrumental or of the sample, $\lambda$ is the wavelength of the X-ray radiation, $D_{hkl}$ is the crystal size or the maximum penetration depth of the x-ray radiation, and $\varepsilon _{hkl}$ is the strain in the direction [hkl]. If the strain is constant in all directions $\varepsilon _{hkl}$ should be replaced by $\varepsilon$. According to the result in Fig. 8b, the strain in the $\alpha$-Al$_2$O$_3$ reference 50 $\mu$m powder was of order $10^{-4}$, which proves that the reference $\alpha$-Al$_2$O$_3$ is strain-free. With these data of broadening, a semi-empirical function of the instrumental broadening with angle is obtained by fitting the obtained reference points to the Cagliotti equation (*cf.* Refs.^2,56^) in the form:

11 $$\begin{aligned} {B}_\text {inst.}^2=U{{\tan }^{2}}\theta +U\tan \theta +W \end{aligned}$$

where the U, V, and W are the fitting parameters, see Fig. 8c. Using the parameter fitting of data to Eq. 11, shown in Fig. 8c, the correct broadening of the pristine and gamma-irradiated $\alpha$-Fe$_2$O$_3$ samples were calculated.

**Figure 9:**
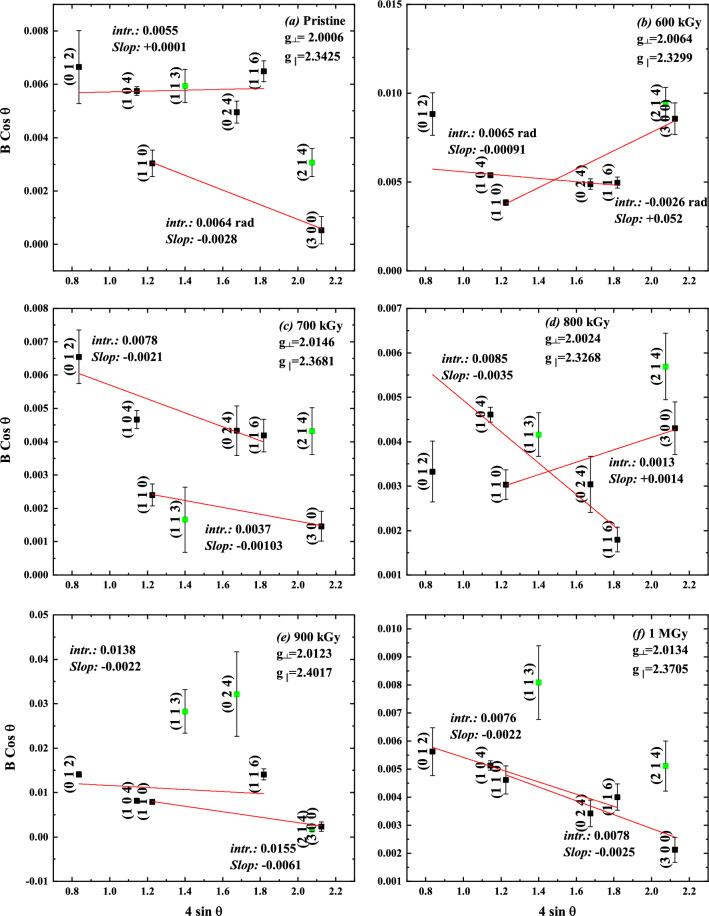
Williamson-Hall plot for the X-ray diffraction of the pristine and gamma-irradiated $\alpha$-Fe$_2$O$_3$ samples. Lines show the fitting parameters to Williamson–Hall Eq. 10.

For the hematite samples, the specimen broadening was used to construct the Williamson–Hall plot in Fig. 9. These results are correlated with the results obtained from the EPR study by inserting the g-values in the label of each sub-figure. For the pristine sample, there are two trends. The, nearly, horizontal slope at the directions comparable to the nanorod axis ([012], [104], [113], [024], and [116]) indicates the absence of strain along that direction, i.e. the crystal free from microstrain in that directions. The strain in the direction perpendicular to the axis of the $\alpha$-Fe$_2$O$_3$ nanorods ([110] and [300]) is negative which indicates compression in the lattice. Both observations are consequences of the growth process from FeOOH into $\alpha$-Fe$_2$O$_3$ as mentioned previously.

The Williamson–Hall plot of the gamma-irradiated $\alpha$-Fe$_2$O$_3$ samples shows fluctuation of the strain in different directions. Upon irradiation at 600 kGy, the stresses along the [110] and [300] crystal directions turn into expansion stress as indicated from the positive slope in Fig. 6b. On the contrary, at 700 kGy of absorbed dose, all crystallographic directions experience compression strain toward the center of mass of the crystal but the lattice size is different as clued from the values of intersections as indicated in Fig. 9b. At 800 kGy of dose, the situation along the [110] and [300] crystal directions is reversed, again, into expansion stress; the compression strain increases along ([012], [104], and [116]) directions.

Further increase of radiation dose to 900 kGy and above cause stabilization of the compression stress in the $\alpha$-Fe$_2$O$_3$ toward a value of -0.0022; i.e., the strain along the [110] and [300] gradually equilibrate with compression strain along other direction after exposure to absorbed doses of 900 kGy and 1000 kGy.

### Correlation among results

#### Correlation between g-value and strain

The nature of the strain, of being compression or expansion stresses, is reflected on the $O_h$-$FeO_6$ octahedron structure distortion through correlation with EPR. The EPR results of the pristine sample show that the values of $g_\perp$ = 2.0006 which is near the free electron case, while the value of $g_\parallel$ is 2.3425. Strain-free crystal may have stable $O_h$-$FeO_6$ octahedron structure as shown in Fig. 6b; however, the existence of two absorptions indicates a Jahn-Teller distortion as indicated in Fig. 6c,d.

Upon irradiation with 600 kGy of absorbed dose, the EPR results showed that the values of $g_\perp$ = 2.0064 and $g_\parallel$ = 2.3299; $g_\perp$ is just above the free electron case while the splitting indicates continuing the distortion in the $O_h$-$FeO_6$ octahedron structure, see Fig. 6c. At 700 kGy of absorbed dose, EPR results reflect lattice distortion by the increased values of $g_\perp$ = 2.0146 and $g_\parallel$ = 2.3681 which is associated with large compression strain, see Fig. 5. EPR results of the 800 kGy sample follow the same trend as the 600 kGy sample where $g_\perp$ is decreased to 2.00237 while $g_\parallel$ decreased to 2.3268 in correlation with the expansion strain. Once the compression strain had developed again at doses 900 kGy and 1 MGy, the values of $g_\perp$ increased to 2.0122 and 2.0134, respectively. The values of $g_\parallel$ also increased to 2.4017 at 900 kGy and 2.3705 at 1 MGy.

This fluctuation in values of the atomic coordination techniques (EPR and XRD) could be understood on the bases of the nature of ionizing radiation effects as a continuous cause having disconnected random responses in the material, the instant of reaching a specific radiation dose would freeze the particular instants of the whole continuous progression of the effect.

#### Correlation between morphology and strain

**Figure 10:**
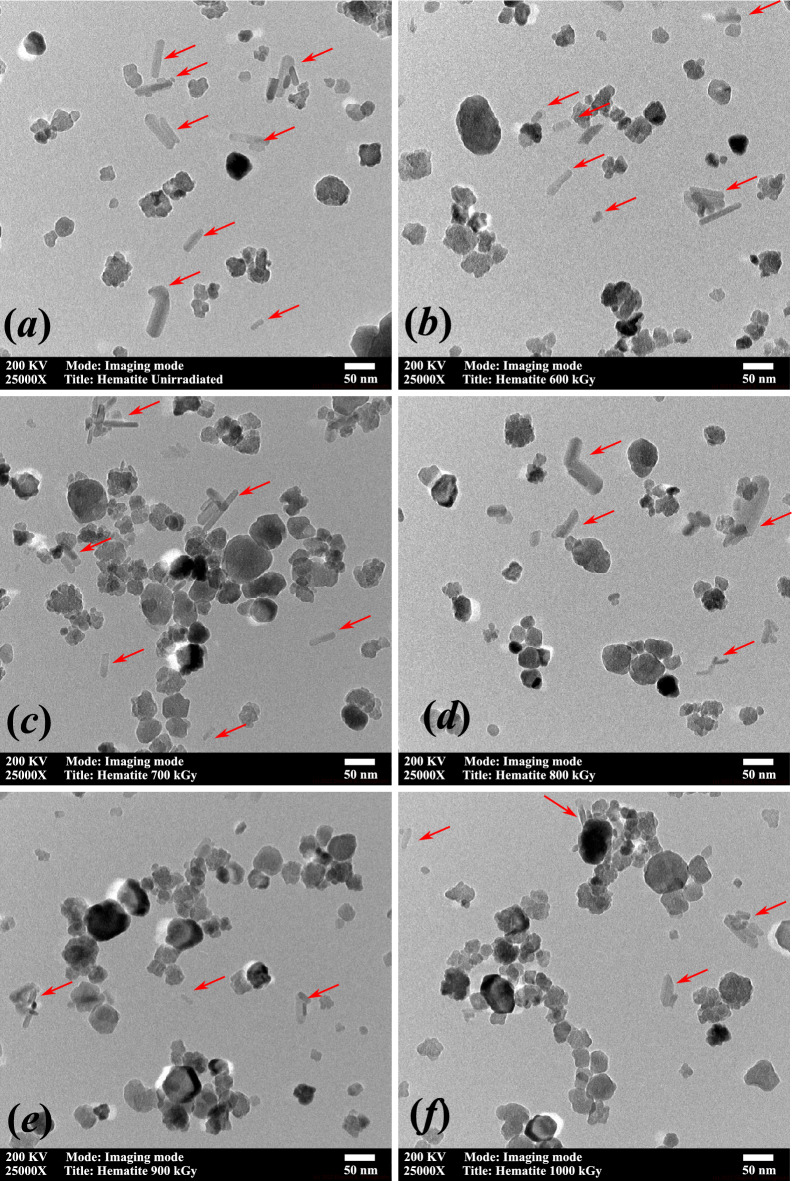
Bright-field TEM image of $\alpha$-Fe$_2$O$_3$ nanorods exposed to different gamma radiation doses. Images are in X25000 magnification. Doses are indicated in the legend of each image. Arrows point to the position of candidate nanorods.

Figure 10a–f shows the TEM images of the $\alpha$-Fe$_2$O$_3$ *powder* before and after exposure to gamma-radiation of different doses. The morphology of the $\alpha$-Fe$_2$O$_3$ nanorods changes with absorbed dose. The pristine sample (Fig. 10a) shows the coexistence of nanorods and buckyball-shaped particles; the proportion of nanorods exceeds 50%. One of the reasons behind the existence of buckyball-shaped structures in the pristine samples is thermal stresses and subsequent thermal relaxation during the preparation phase. Upon irradiation, the statistical proportion of nanorods’ shape experiences a change in morphology in which the proportion of nanorods decreases relative to other buckyball-shaped particles down into less than 10% nanorods after exposure to 900 kGy of gamma radiation. Despite the fluctuation of the strain and g-value with radiation dose, the TEM investigation showed that the nanorods’ morphology consistently transforms into buckyball-shaped particles as the dose increases.

## Conclusion

The thermodynamics of the preparation parameters affects the $\alpha$-Fe$_2$O$_3$ crystal morphology through the diversity of the growth rate along different crystal directions. In particular, the preferential growth along the [001] direction (the (001) basel plane) is attributed to the higher (001) plane affinity to attract Fe-O species than other crystal planes under the used condition of microwave-assisted hydrothermal synthesis.

The XRD analysis showed that the [012], [104], [113], and [024] crystallographic directions of pristine $\alpha$-Fe$_2$O$_3$ nanorods are strain-free. However, $\alpha$-Fe$_2$O$_3$ nanorods crystallize in a manner that makes the [110] and [300] rhombohedral lattice directions have a compression strain. The corresponding stresses are attributed to the accelerated growth along [001] direction; which is perpendicular to the [110] and [300] lattice directions. The formed nanorods should preserve their shape under normal thermodynamic conditions. The EPR showed the splitting associated with a distorted octahedral crystal field in correlation with the compression strain. Its TEM image showed also a large proportion of nanorods compared to collapsed buckyball-shaped particles.

Upon irradiation, the energy deposition within the crystal may facilitate the relaxation of the compression strain or cause additional stresses along an axis perpendicular to the rod axis. Compression strain along the [012], [104], [113], and [024] crystallographic directions persist with increasing radiation dose. These phenomena were investigated using both XRD and EPR techniques which showed a direct correlation between the $O_h$-$FeO_6$ octahedron structure distortion and the strain of being compression or expansion, reaching an equilibration phase between 900 kGy and 1 MGy of absorbed dose.

The TEM investigation showed that the fluctuation in the strain and their associated lattice distortion are correlated to the particle morphology in which the continuous increase in radiation dose and consequently the increase in radiation energy deposition would transform a proportion of the rod-shaped particle into buckyball-shaped particles. This full relaxation of $\alpha$-Fe$_2$O$_3$ nanorods lattice may happen after samples had been exposed to 1000 kGy of absorbed dose where the strain becomes between $-0.0025$ and $-0.0022$ along all crystallographic planes where the values of $g_\perp$ and $g_\parallel$ reached 2.0134, and 2.3705, respectively. The results threw doubt on the stability of $\alpha$-Fe$_2$O$_3$ nanorods for application in different environments including the high radiation environment and during electrochemical long-term operation.

## Data Availability

All data generated or analysed during this study are included in this published article.
